# Account of Diseases in an Eastern District of London, from the 20th of July, to the 20th of August

**Published:** 1799-09

**Authors:** 


### STATE OF DISEASES IN LONDON.

*Account of Diseases in an Eastern District of London, from the 20th of July, to the 20th of August.*

ACUTE DISEASES.

| | No. of Cases. |
|---|---|
| Typhus | 2 |
| Pneumonia | 2 |
| Catarrh | 1 |
| Acute Rheumatism | 3 |
| Variolæ | 1 |

CHRONIC DISEASES.

| | No. of Cases. |
|---|---|
| Cough | 6 |
| Dyspnœa | 7 |
| Asthma | 3 |
| Pleurodyne | 3 |
| Phthisis Pulmonalis | 7 |
| Hydrothorax | 2 |
| Ascites | 5 |
| Vertigo | 3 |
| Paraplegia | 1 |
| Epilepsy | 2 |
| Syncope | 1 |
| Palpitatio | 1 |
| Dyspepsia | 7 |
| Gastrodynia | 5 |
| Diarrhœa | 10 |
| Enterodynia | 4 |
| Dysenteria | 3 |
| Colica | 1 |
| Colica Pictonum | 3 |
| Menorrhagia | 2 |
| Amenorrhœa | 3 |
| Chlorosis | 4 |
| Prolapsus Vaginæ | 1 |
| Hæmorrhois | 3 |
| Enuresis | 2 |
| Calculus | 1 |
| Dysuria | 3 |
| Nephritis | 1 |
| Hysteria | 3 |
| Hypochondriasis | 2 |
| Lepra | 1 |
| Herpes | 4 |
| Exostosis | 3 |
| Lumbago | 2 |
| Sciatica | 3 |
| Chronic Rheumatism | 13 |

PUERPERAL DISEASES.

| | |
|---|---|
| Ephemera | 2 |
| Milk Fever | 3 |
| Menorrhagia lochialis | 2 |

INFANTILE DISEASES.

| | |
|---|---|
| Ophthalmia | 2 |
| Aphthæ | 3 |
| Dentitio | 2 |
| Tabes Mesenterica | 1 |
| Vermes | 3 |

There has been nothing in the state of disease during the last month, that deserves any particular attention. The state of the weather, however unfavourable it may prove to the vegetable, does not seem to have produced much derangement of the animal economy. The bowels have been the principal seat of complaint. A few instances of slight dysentery, with a larger number of diarrhœa seem to constitute the list of diseases at present prevailing.

